# Proteomics Study of Peripheral Blood Mononuclear Cells (PBMCs) in Autistic Children

**DOI:** 10.3389/fncel.2019.00105

**Published:** 2019-03-19

**Authors:** Liming Shen, Chengyun Feng, Kaoyuan Zhang, Youjiao Chen, Yan Gao, Junyan Ke, Xinqian Chen, Jing Lin, Cuihua Li, Javed Iqbal, Yuxi Zhao, Weibin Wang

**Affiliations:** ^1^College of Life Science and Oceanography, Shenzhen University, Shenzhen, China; ^2^Maternal and Child Health Hospital of Baoan, Shenzhen, China; ^3^Xiang Ya Changde Hospital, Changde, China; ^4^School of Art, Shenzhen University, Shenzhen, China

**Keywords:** autism, biomarkers, iTRAQ, peripheral blood mononuclear cells, proteomics

## Abstract

Autism is one of the most common neurological developmental disorder associated with social isolation and restricted interests in children. The etiology of this disorder is still unknown. There is neither any confirmed laboratory test nor any effective therapeutic strategy to diagnose or cure it. To search for biomarkers for early detection and exploration of the disease mechanisms, here, we investigated the protein expression signatures of peripheral blood mononuclear cells (PBMCs) in autistic children compared with healthy controls by using isobaric tags for relative and absolute quantitation (iTRAQ) proteomics approach. The results showed a total of 41 proteins as differentially expressed in autistic group as compared to control. These proteins are found associated with metabolic pathways, endoplasmic reticulum (ER) stress and protein folding, endocytosis, immune and inflammatory response, plasma lipoprotein particle organization, and cell adhesion. Among these, 17 proteins (13 up-regulated and four down-regulated) are found to be linked with mitochondria. Eight proteins including three already reported proteins in our previous studies were selected to be verified. Five already reported autism associated pro-inflammatory cytokines [interferon-γ (IFN-γ), interleukin-1β (IL-1β), IL-6, IL-12, and tumor necrosis factor-α (TNF-α)] were detected in plasma by enzyme-linked immunosorbent assay (ELISA) analysis. The results were consistent with proteomic results and reports from previous literature. These results proposed that PBMCs from autistic children might be activated, and ER stress, unfolded protein response (UPR), acute-phase response (APR), inflammatory response, and endocytosis may be involved in autism occurrence. These reported proteins may serve as potential biomarkers for early diagnosis of autism. More specifically, simultaneous detection of three proteins [complement C3 (C3), calreticulin (CALR), and SERPINA1] in the plasma and PBMCs could increase the authenticity of detection.

## Introduction

Autism spectrum disorder (ASD) is a group of developmental neurological disorders characterized by various impairments in communication and social interactions as well as restricted interests and repetitive behaviors (Faras et al., 2010). Based on DSM-IV (Fourth Edition of the Diagnostic and Statistical Manual of Mental Disorders), it can be referred as childhood autism, Asperger syndrome, Rett syndrome (RTT), pervasive developmental disorders and other childhood disintegrative disorders. Among these, autism is the most severe and classical disorder (Rangasamy et al., 2013).

Over the past 20 years, the prevalence of ASD has been continuously increasing all over the world. As the etiology and pathogenesis have not yet been completely elucidated, ASD diagnosis remained a behavioral or symptomatic rather than a molecular diagnosis. Due to lack of information regarding molecular mechanisms of the disease, no effective therapeutic approach is currently available. However, the previous studies suggest that early behavioral intervention is associated with normalized patterns of brain activity. Therefore, early diagnosis of this disease is crucial and provides interventions that can significantly influence children’s outcomes (Dawson et al., 2012; Dawson, 2013). Thus, intensive research has been made to identify biological markers for disease management and early diagnosis. Meanwhile, the etiology underlying ASD at the molecular, cellular and systems level remained elusive and needs to be further clarified.

The etiology of ASD is commonly described as a genetic predisposition combined with an environmental impact (Chaste and Leboyer, 2012). It is estimated that 400–1,000 genes are likely to be involved in autism (Masi et al., 2017a). However, it is a heterogeneous disorder with complex genetic basis. Only 10%–15% of ASD cases may be etiologically related to known genetic disorders and specific genes still need to be associated with autism (Folstein and Rosen-Sheidley, 2001). No single gene can be considered “causal” for more than 1% of cases of idiopathic autism (Hu, 2013). Interestingly, recent studies demonstrated that the large set of ASD genes converges on a smaller number of key pathways and developmental stages of the brain. For example, many ASD genes are known to regulate brain development and/or synapse function (Krishnan et al., 2016). Thus, it will be quite interesting to study the ASD from the protein perspective.

Genomic research has increased the importance of studying ASD-related differentially expressed genes from peripheral tissues (blood and peripheral blood cells) and postmortem brains of ASD subjects (Baron et al., 2006; Hu et al., 2006; Nishimura et al., 2007; Kuwano et al., 2011). However, for a variety of reasons, ASD related brain studies are very difficult, therefore, performed at a limited level. Interestingly, a moderate correlation of gene expression between peripheral blood cells and brain tissue in humans has been reported, supporting the usefulness of peripheral blood cells in gene expression studies for psychiatric research (Sullivan et al., 2006). In addition, infection and immune responses have already been implicated in ASD. Immunity and inflammation-associated lymphoblastoid cell lines (LCLs) have been used by a number of groups to study gene expression in ASD (Hu, 2014). Besides, monocytes in blood and microglia in the brain have very similar transcriptomes, while abnormalities in microglia have been observed in ASD brains (Vargas et al., 2005; Morgan et al., 2010). Dysfunction in peripheral blood mononuclear cells (PBMCs) could result in long-term immune alterations in ASDs has already been reported (Ashwood et al., 2006; Enstrom et al., 2010), and the alterations of mRNA expressions in PBMCs obtained from ASD subjects has also been determined (Glatt et al., 2012). Therefore, PBMCs may represent a useful tool to investigate systemic neurochemical changes in neurodevelopmental diseases. Keeping in mind the above discussion, there must be some protein changes that can be recorded which might prove as potential biomarkers and can be used in ASD diagnosis. In the present study, we have made an attempt to search for protein based potential biomarkers for the diagnosis of autism and tried to further clarify the molecular mechanisms of this disease by using isobaric tags for relative and absolute quantitation (iTRAQ) quantitative proteomics approach in autistic children.

## Materials and Methods

### Subjects Population

Twenty four male and six female autistic children (2–6 years old) were recruited along with gender and age-matched controls from Populations and Family Planning Hospital of Baoan. The diagnosis of autism was based on the criteria of autistic disorders as defined in the DSM-IV by a child neuropsychiatrist. There were no significant differences in weight, height or body mass index (BMI) between the case and control groups. The experiments were performed with the written consent of the caretakers of the children under observation according to the guidelines of this hospital (Feng et al., 2017; Shen et al., 2018).

### Protein Sample Preparation

**Figure 1:**
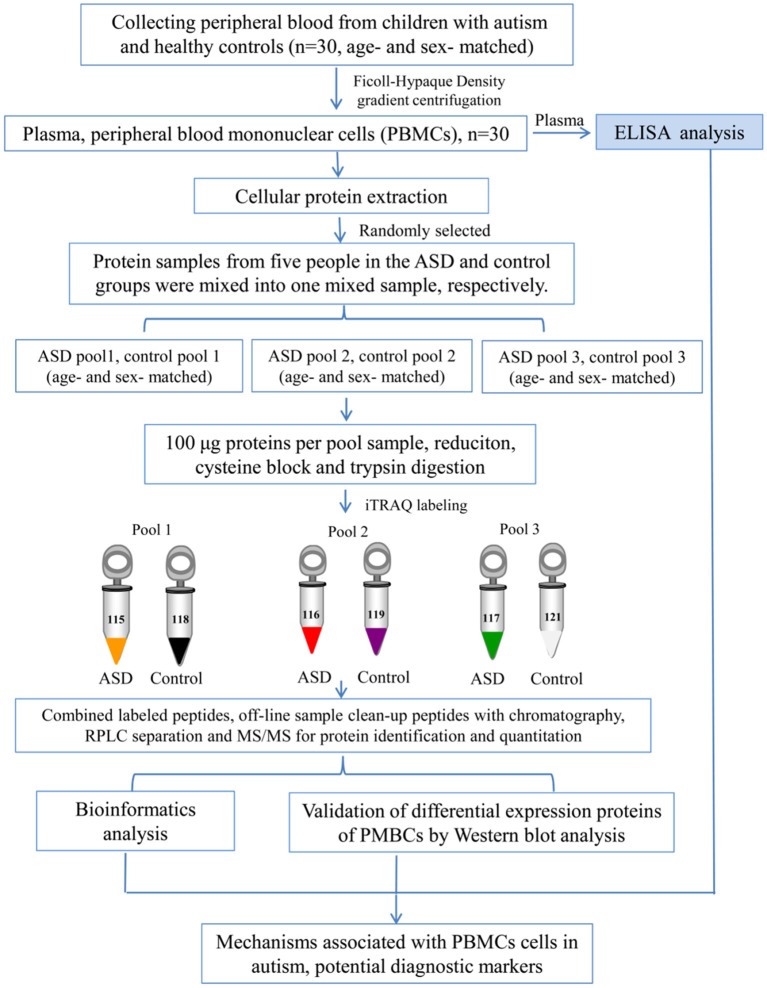
Workflow used in this study.

Blood samples (5 ml) were collected in EDTA-coated plastic tubes in the morning while the subjects were in the fasting state. PBMCs were separated by density gradient centrifugation using Ficoll-Hypaque (Sigma-Aldrich, St. Louis, MO, USA) as previously described (Nilsson et al., 2008). The cells were lysed in lysis buffer (7 M urea, 2 M thiourea, 4% (w/v) CHAPS, 40 mM dithiothreitol (DTT), and 40 mM Tris base), sonicated 10 times for 5 s with 10 s pause interval in an ice-water bath, and centrifuged at 13,800×*g*, at 4°C for 60 min (Heraeus Fresco 17 Centrifuge, Thermo Fisher Scientific Inc., Waltham, MA, USA). The supernatant was taken and stored at −80°C until use. The protein concentrations were determined and optimized by Bradford assay. An overview of the workflow used in this study is shown in Figure 1.

### iTRAQ Labeling and High-pH RPLC Fractionation

The iTRAQ analysis was performed according to the previous study (Sun et al., 2015; Shen et al., 2017; Zhao et al., 2017; Iqbal et al., 2018). As shown in Figure 1, protein samples were analyzed in three biological replicates. In each repetition, we randomly selected five children with ASD from the total recruitment of autistic children, including one girl and four boys, and then selected age and gender matched healthy controls. Equal amounts of plasma were taken from autistic and control children and mixed to form autistic and control pool samples, respectively. Protein extraction (100 μg) from each sample was reduced, alkylated and digested with trypsin (Promega, Madison, WI, USA) at a ratio of 1:30 at 37°C for overnight, and then labeled with iTRAQ reagents (AB Sciex, Foster City, CA, USA). Three samples from autistic children were labeled with iTRAQ tags 115, 116, 117, and the healthy controls with 118, 119 and 121 tags, respectively. After labeling, these were incubated at room temperature for 1 h, and then mixed and lyophilized. The dried samples were reconstituted in 100 μL of double-distilled water and injected into the Agilent [high-performance liquid chromatography (HPLC); Agilent Technologies, Santa Clara, CA, USA] with a high pH RP (reverse phase) column (Durashell, C18, 250 mm × 4.6 mm, 5 μm; Bonna-Agela Technologies Inc., Wilmington, DE, USA). Peptides were eluted and combined into 10 groups and lyophilized (Shen et al., 2017; Zhao et al., 2017).

### NanoLC-MS/MS (Mass Spectrometry) Analysis

An Ultra 2D Plus nanoflow HPLC (Eksigent Inc., Dublin, CA, USA) coupled with Triple TOF 5600 system (AB Sciex) was used for the analytical separation of peptides (Sun et al., 2015; Shen et al., 2017; Zhao et al., 2017; Iqbal et al., 2018). Labeled peptides were loaded on the column (ChromXP C18, 3 μm, 350 μm × 0.5 mm, Eksigent) with trapping and desalting carried out at 5 μL/min for 5 min using mobile phase A (2% acetonitrile, 0.1% formic acid). Analytical separation was carried out with ChromXP C18 (3 μm, 75 μm × 150 mm, Eksigent) at a flow rate of 350 nL/min. The elution gradient was run using mobile phase A and B (98% acetonitrile, 0.1% formic acid). Gradient of mobile phase and run time are shown in Supplementary Table S1. The mass spectrometric data was acquired in positive ion mode, with a 2.4-kV ion spray voltage, 30-psi curtain gas, 5-psi nebulizer gas, and an interface heater temperature of 150°C. The scan scope for TOF-mass spectrometry (MS) is 350–1,500 m/z, and peptides with +2 to +5 charge states were selected for MS/MS analysis (mass range of 100–1,500 m/z). Survey scans were acquired in 0.25 s and a maximum of 30 product ion scans were run with accumulation time of 80 ms for each product ion scan. Smart information-dependent acquisition (IDA) was activated with automatic collision energy and automatic MS/MS accumulation.

### Database Search and iTRAQ Quantification

Protein identification and quantification were performed using ProteinPilot v4.5 (AB Sciex) with the Paragon Algorithm against the uniprot “complete proteome” human proteins database. To reduce false-positive identification results, false discovery rate (FDR) less than 1% was required for the identification of proteins (Tambor et al., 2016). The ProteinPilot Descriptive Statistics Template was used for estimation of FDR associated with quantification results (Tambor et al., 2016). Based on a 95% confidence level, at least one unique peptide per protein group was required for identifying proteins, and two quantified peptides were required for quantifying proteins. The cutoff values of 1.5-fold for up-regulated and 0.67-fold for down-regulated proteins, *P*-value <0.05, and consistent change in ratios in all cross comparisons (115:118, 115:119, 115:121; 116:118, 116:119, 116:121; 117:118, 117:119, 117:121), were established for significantly differentially expressed proteins between autistic patients and controls. This significant change was further determined through the conversion of log_2_ ratios (include ASD vs. control, control vs. control), followed by data normalization, and statistical *t*-test, and cluster analysis by using OMICSBEAN online tools^1^.

### Bioinformatics Analysis

The differentially expressed proteins were loaded to DAVID (Database for Annotation, Visualization and Integrated Discovery^2^) database for biological process (BP), cellular component (CC), and Kyoto Encyclopedia of Genes and Genomes (KEGG) pathways analysis. Protein-protein interaction (PPI) networks were analyzed by using OMICSBEAN^1^ database. Functional interaction network analysis was performed using ClueGO cytoscape plugin (Bindea et al., 2009). GO (gene ontology) categories and pathways searched include BPs, molecular functions (MFs), Kyoto Encyclopedia of Genes and Genomes (KEGG), REACTOME and Wiki pathways.

### Western Blot Analysis

To validate quantitative proteomic results, protein samples were selected randomly from the cases and controls with gender and age-matched (*n* = 5). The samples were analyzed by Western blotting using specific antibodies. Proteins (20 μg/lane) were separated by SDS-PAGE on 12% polyacrylamide gels, and then transferred to polyvinylidene fluoride (PVDF) membranes (Millipore, Billerica, MA, USA), and blots were blocked with 5% skimmed-dried milk in phosphate-buffered saline (PBS: 137 mM NaCl, 10 mM phosphate buffer, and 2.7 mM KCl, pH 7.4) for 1 h and then washed with PBS containing 0.2% (v/v) tween 20 (PBST) and further incubated for overnight at 4°C using primary antibodies against, alpha-1-antitrypsin (SERPINA1), ATP synthase subunit alpha, mitochondrial (ATP5A1), ATP synthase subunit beta, mitochondrial (ATP5B), calreticulin (CALR), complement C3 (C3), cytochrome b-c1 complex subunit 2, mitochondrial (UQCRC2), malate dehydrogenase, mitochondrial (MDH2), and flotillin-1 (FLOT1), at optimized dilutions with PBST. All primary antibodies were purchased from Abcam Inc (Cambridge, MA, USA). After washing with PBST, the blots were then incubated in 1:5,000-diluted horseradish peroxidase (HRP)-conjugated secondary antibody (Abmart Inc, Shanghai, China) for 2 h at room temperature. The membranes were washed three times each for 5 min in PBST and developed with enhanced chemiluminescence (ECL) kit (Pierce ECL detection kit, Thermo Fisher Scientific Inc, Rockford, USA). Immunoreactive signals were detected with a Kodak Image Station 4000MM imaging system (Carestream Health Inc., Rochester, NY, USA). Quantitative analysis of protein bands was performed using Quantity One analysis software (Bio-Rad, USA).

### ELISA Analysis

Plasma from individual autistic patients and controls was used to carry out the quantitative detection of SERPINA1 and CALR using a commercially available enzyme-linked immunosorbent assay (ELISA) kit (Uscn Life Science Inc., Cloud-Clone Corp., USA) as per instructions from the manufacturer. Five pro-inflammatory cytokines i.e., interferon-γ (IFN-γ) interleukin-1β (IL-1β), IL-6, IL-12, and tumor necrosis factor-α (TNF-α) were also measured using ELISA kits (Neobioscience Technology Company, Guangdong, China) following the manufacturer’s instructions. The ELISA results were normalized to total protein concentrations.

### Statistical Analysis

For Western blot and ELISA analysis, statistically significant difference was determined by *t*-test with GraphPad Prism Software (GraphPad Software, Inc., San Diego, CA, USA). *P* value < 0.05 was considered as statistically significant.

## Results

### iTRAQ Comparative Proteomics Results

**Figure 2:**
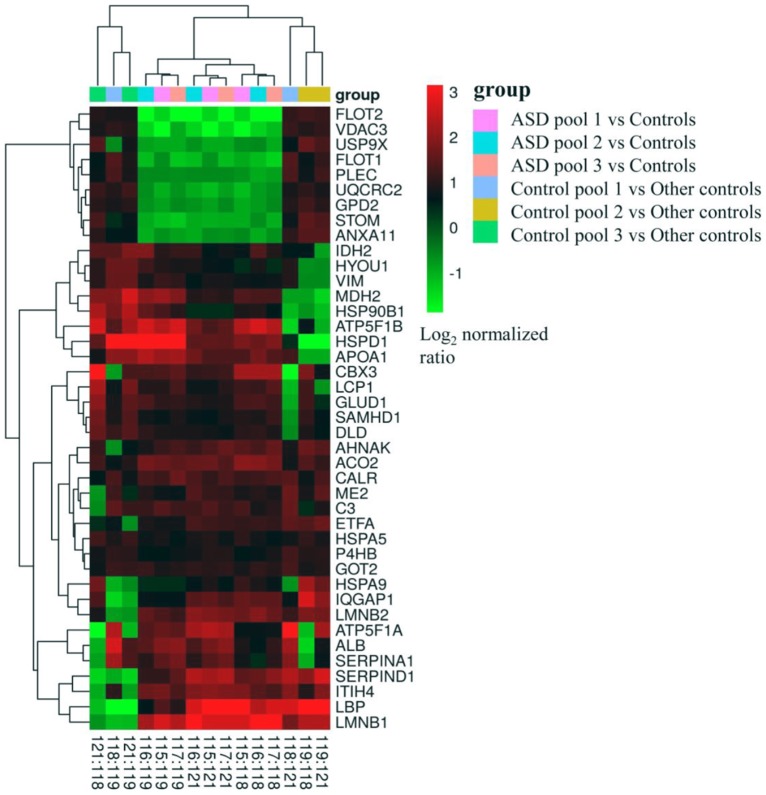
Cluster map the differentially expressed proteins in all cross comparisons. Three mixed samples from autistic children were labeled with isobaric tags for relative and absolute quantitation (iTRAQ) tags 115, 116, 117, and the healthy controls with 118, 119 and 121 tags, respectively.

By iTRAQ analysis, a total of 2,816 proteins were successfully identified with quantification of 2,117 proteins (Supplementary Table S2). Forty-one proteins were identified as differentially expressed proteins between autistic children and healthy controls (Table 1, Figure 2, and Supplementary Table S3). Among these, 32 proteins were significantly up-regulated and nine down-regulated. Interestingly, three proteins, i.e., C3, CALR, and SERPINA1 were also identified as differentially expressed proteins in the plasma of autistic children in our previous study (Shen et al., 2018). The expression trends of C3 and SERPINA1 found here are consistent with the previous study. However, the level of CALR is significantly up-regulated in this study and down-regulated in the previous study (Shen et al., 2018).

**Table 1:** The differentially expressed proteins identified in peripheral blood mononuclear cells (PBMCs) from children with autism and healthy controls.

No.	Protein name	Accession #	Gene name	Peptides (95%)	% Cov	Fold change^c^	Pval (FDR correction)
1	**60 kDa heat shock protein, mitochondrial (+)^a, b^**	P10809	HSPD1	59	68.9	4.67	0.013902986
2	**78 kDa glucose-regulated protein** (+)	P11021	HSPA5	49	64.8	2.08	2.24E-06
3	**Aconitate hydratase, mitochondrial** (+)	Q99798	ACO2	14	42.3	3.95	9.25E-10
4	Alpha-1-antitrypsin* (+)	P01009	SERPINA1	15	58.1	2.53	0.002748253
5	Annexin A11 (−)	P50995	ANXA11	18	30.5	0.55	2.69E-07
6	Apolipoprotein A-I (+)	P02647	APOA1	15	56.6	3.78	0.000365353
7	**Aspartate aminotransferase, mitochondrial** (+)	P00505	GOT2	7	40	2.38	3.80E-06
8	**ATP synthase subunit alpha, mitochondrial** (+)	P25705	ATP5A1	61	65.6	2.94	0.019869459
9	**ATP synthase subunit beta, mitochondrial** (+)	P06576	ATP5B	67	83.4	4.66	5.11E-05
10	Calreticulin* (+)	P27797	CALR	26	58	2.93	8.09E-06
11	Chromobox protein homolog 3 (+)	Q13185	CBX3	10	50.8	3.31	3.02E-05
12	Complement C3* (+)	P01024	C3	38	43.8	2.69	1.46E-05
13	**Cytochrome b-c1 complex subunit 2, mitochondrial** (−)	P22695	UQCRC2	26	64	0.52	0.002836642
14	Deoxynucleoside triphosphate triphosphohydrolase SAMHD1 (+)	Q9Y3Z3	SAMHD1	14	35.1	2.16	6.94621E-05
15	**Dihydrolipoyl dehydrogenase, mitochondrial** (+)	P09622	DLD	12	48.5	2.17	0.000101238
16	**Electron transfer flavoprotein subunit alpha, mitochondrial** (+)	P13804	ETFA	7	49	2.49	4.02001E-08
17	Endoplasmin (+)	P14625	HSP90B1	31	54.9	1.97	0.01778616
18	**Erythrocyte band 7 integral membrane protein** (−)	P27105	STOM	78	77.4	0.48	2.63E-07
19	Flotillin-1 (−)	O75955	FLOT1	20	61.4	0.45	0.003135849
20	Flotillin-2 (−)	Q14254	FLOT2	17	55.6	0.30	1.13E-04
21	**Glutamate dehydrogenase 1, mitochondrial** (+)	P00367	GLUD1	17	44.6	2.50	5.48E-05
22	**Glycerol-3-phosphate dehydrogenase, mitochondria**l (−)	P43304	GPD2	34	61.1	0.58	0.001016776
23	Heparin cofactor 2 (+)	P05546	SERPIND1	5	18.2	4.42	0.001552831
24	Hypoxia up-regulated protein 1 (+)	Q9Y4L1	HYOU1	28	48.4	1.98	0.001809154
25	Inter-alpha-trypsin inhibitor heavy chain H4 (+)	Q14624	ITIH4	10	26.5	3.90	6.04E-06
26	**Isocitrate dehydrogenase [NADP], mitochondrial** (+)	P48735	IDH2	20	60	2.39	0.001550526
27	Lamin-B1 (+)	P20700	LMNB1	20	54.3	7.74	2.24E-06
28	Lamin-B2 (+)	Q03252	LMNB2	10	49.7	3.77	1.24E-06
29	Lipopolysaccharide-binding protein (+)	P18428	LBP	7	33.9	5.48	0.008200263
30	**Malate dehydrogenase, mitochondrial** (+)	P40926	MDH2	16	55	2.95	0.002301777
31	**NAD-dependent malic enzyme, mitochondrial** (+)	P23368	ME2	20	64	2.34	8.07E-06
32	Neuroblast differentiation-associated protein AHNAK (+)	Q09666	AHNAK	23	32.4	3.08	6.08E-06
33	Plastin-2 (+)	P13796	LCP1	41	55	2.42	1.90E-04
34	Plectin (−)	Q15149	PLEC	65	46.8	0.60	0.006498826
35	Probable ubiquitin carboxyl-terminal hydrolase FAF-X (−)	Q93008	USP9X	11	22	0.51	7.70E-05
36	Protein disulfide-isomerase(+)	P07237	P4HB	36	58.9	1.99	5.08E-06
37	Ras GTPase-activating-like protein IQGAP1 (+)	P46940	IQGAP1	37	46.7	2.80	6.60E-06
38	Serum albumin (+)	P02768	ALB	42	64.5	2.91	0.004346685
39	**Stress-70 protein, mitochondrial** (+)	P38646	HSPA9	19	54.2	1.85	3.42E-05
40	Vimentin (+)	P08670	VIM	28	62	2.07	0.0011167
41	**Voltage-dependent anion-selective channel protein 3** (−)	Q9Y277	VDAC3	36	68.2	0.35	0.000430871

^a^(+), proteins increased in abundant; (−), proteins decreased in abundant. ^b^The differentially expressed proteins belong to mitochondrial proteins are indicated in bold. ^c^Fold change, *P* < 0.05 vs. the control. “*” The protein has been identified as differentially expressed protein in the blood of autistic patients in our previous study (Shen et al., 2018).

### Bioinformatics Analysis of the Differentially Expressed Proteins

**Figure 3:**
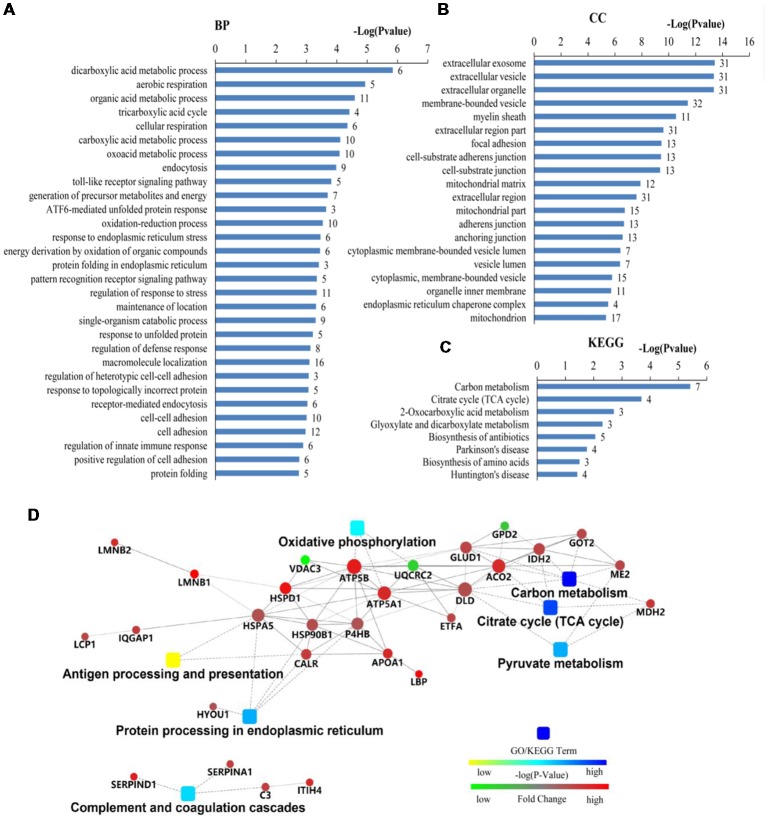
Bioinformatics analysis of 41 differentially expressed proteins in peripheral blood mononuclear cells (PBMCs) from autistic children and healthy subjects. **(A)** The top 30 ranking of biological process (BP) based on gene ontology (GO). **(B)** The top 20 ranking of cellular components (CCs) based on GO. **(C)** By using DAVID analysis, the Kyoto Encyclopedia of Genes and Genomes (KEGG) signal transduction pathways are associated with these proteins. **(D)** Protein-protein interaction (PPI) networks of differentially expressed proteins. The networks analysis was carried out by using OMICSBEAN. **(A–C)** Number of proteins associated with each category and *P*-value for gene-enrichment analysis is shown on the right of each term bar.

The differentially expressed proteins were analyzed by different databases. By using DAVID database, BP analysis showed that these proteins are mainly related to dicarboxylic acid metabolic process, erobic respiration, cellular respiration, organic acid metabolic process, tricarboxylic acid cycle (TCA cycle), carboxylic acid metabolic process, response to endoplasmic reticulum (ER) stress, protein folding, response to unfolded protein, protein folding in ER, ATF6-mediated unfolded protein response (UPR), toll-like receptor (TLR) signaling pathway, exocytosis, receptor-mediated endocytosis, cell adhesion, regulation of response to stress, and regulation of immune system process, et cetera (Figure 3A). CC analysis showed that these proteins are integral part of extracellular exosome, extracellular vesicle, extracellular organelle, focal adhesion, myelin sheath, mitochondrion, cell-substrate adherens junction, cell-substrate junction, adherens junction, anchoring junction, et cetera (Figure 3B). Pathway analyses indicated that they are involved in the carbon metabolism, citrate cycle (TCA cycle), 2-Oxocarboxylic acid metabolism, glyoxylate and dicarboxylate metabolism, biosynthesis of antibiotics, Parkinson’s disease, biosynthesis of amino acids, and Huntington’s disease (Figure 3C). The BPs and pathways related to these proteins obtained from DAVID database are listed in Supplementary Table S4, including the count, percent (%) of associated genes, *P*-values, and gene names related to the different GO categories and pathways. Protein-protein interactions (PPIs) obtained from OMICSBEAN database is shown in Figure 3D. Twenty-nine proteins interact with each other in a protein interaction network, similar to pathway analysis by DAVID database. Some metabolic processes and ER related cascades are linked to these proteins. Moreover, complement and coagulation processes are also involved.

**Figure 4:**
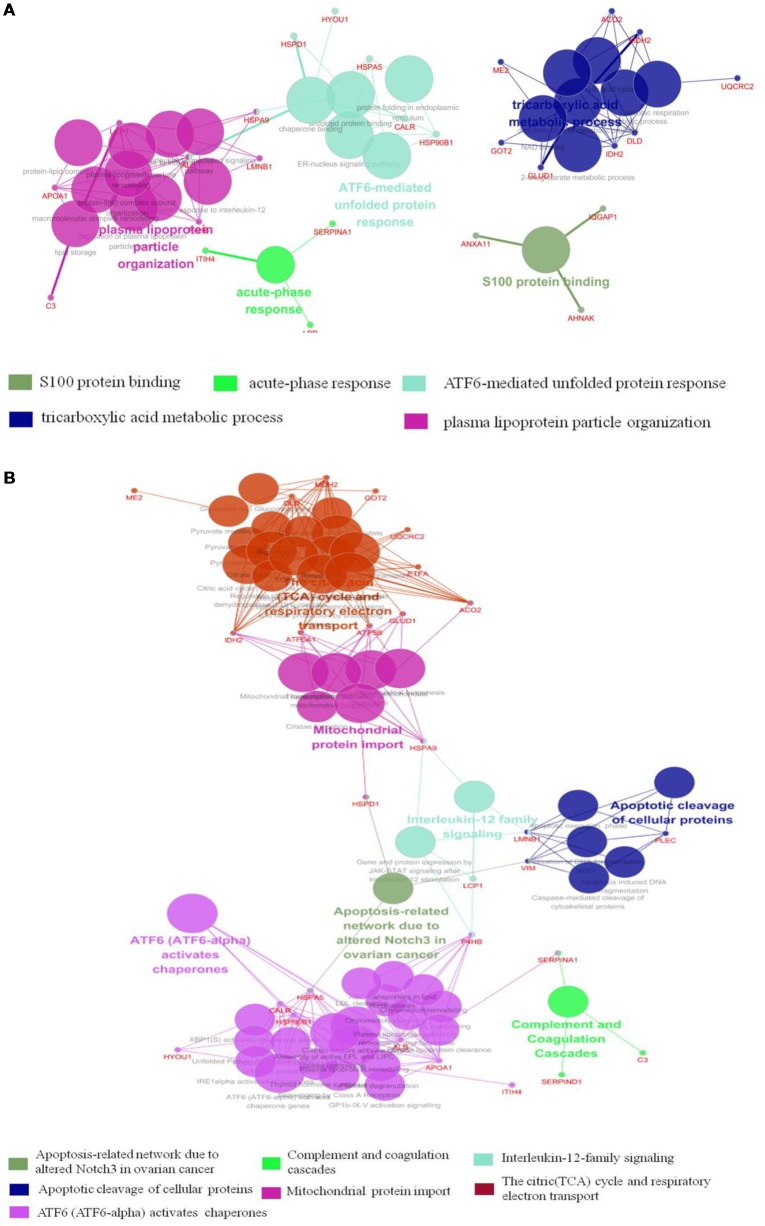
Functional interaction network analysis was performed using ClueGO cytoscape plugin.** (A)** The differentially expressed 41 proteins were mapped to the GO categories (BPs and molecular function, MF). **(B)** The differentially expressed proteins were mapped to KEGG pathway, REACTOME pathway and Wiki pathway. Panels **(A,B)** are separately uploaded in the Supplementary Figure S1.

The results of Cytoscape analysis are overlapping with DAVID and OMICSBEAN analysis. The BPs and MFs associated with these proteins include cellular respiration, regulation of plasma lipoprotein particle levels, tricarboxylic acid metabolic process, protein folding in ER, acute-phase response (APR), S100 protein binding, IL-12-mediated signaling pathway, et cetera (Figure 4A). Pathway analysis showed that these proteins are mainly involved in citrate cycle (TCA cycle), mitochondrial protein import, mitochondrial biogenesis, ATF6 (ATF6-alpha) activates chaperones, apoptotic cleavage of cellular proteins, complement and coagulation cascades, and IL-12 family signaling, et cetera (Figure 4B).

**Figure 5:**
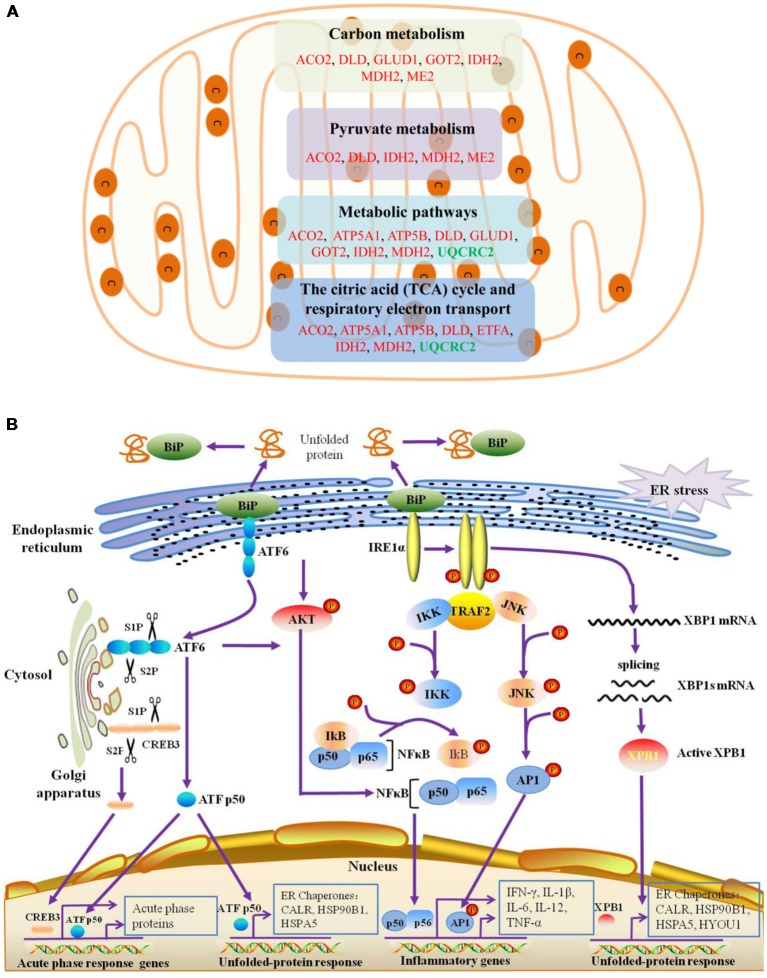
Mitochondrial, endoplasmic reticulum (ER)-stress, unfolded protein response (UPR), inflammatory response and acute phase response pathways are associated with differentially expressed proteins.** (A)** The differentially expressed proteins belonged to mitochondrial proteins are mainly divided into four categories, which are associated with different pathways. **(B)** ER-stress, UPR, acute phase response pathways are shown. ATF6 and IRE1α pathways may be involved in the unfolded-protein response and inflammatory response of PBMCs in autistic children. Upon dissociation from BiP in response to ER stress, ATF6 translocates to the Golgi apparatus, where it is cleaved by the proteases site-1 protease (S1P) and S2P. This process results in the release of a functional (bZIP-containing) fragment of ATF6 into the cytosol. This fragment then migrates to the nucleus and activates transcription. In response to ER stress, IRE1α autophosphorylates, thereby activating its RNase activity and splices XBP1 mRNA to spliced XBP1 mRNA, which codes for a transcription factor XBP1s that translocates to the nucleus and regulates genes involved in UPR and ER-associated degradation (ERAD). In addition, ATF6 can also trigger NFκB-mediated inflammation *via* Akt kinase phosphorylation (pAkt). IRE1α induces the expression of inflammatory genes through the activation of the JNK-AP1 and NF-κB-Iκ-B pathways. Moreover, active ATF6 and CREB3 can induce the transcription of acute phase response genes. CREB3 is cleaved by the Golgi resident proteases S1P and S2P. This protein fragment then transits from the cytosol to the nucleus where it activates transcription of target genes. The differentially expressed proteins associated with ER stress, protein folding in ER, unfolded protein binding, ATF6 activates chaperones, XBP1s activates chaperones, IRE1alpha activates chaperones, CREB3 factors activate genes, and acute inflammatory response, are listed in Supplementary Tables S4–S6.

The BPs and MFs related to these proteins and obtained from Cytoscape are given in Supplementary Table S5, and pathways related to them are presented in Supplementary Table S6. GO Term, ontology source, term and group *P*-values, GO Groups, percent (%) of associated genes, gene number, and gene names associated with the differentially expressed proteins are listed in these two tables. Of note, 17 proteins, i.e., ACO2, ATP5A1, ATP5B, DLD, ETFA, GLUD1, GOT2, HSPA5, HSPA9, HSPD1, IDH2, MDH2, ME2, STOM, TUFM, UQCRC2, and VDAC3 belong to mitochondria. Interestingly, except for GPD2, UQCRC2, and VDAC3, all of the other proteins were up-regulated in the autistic children compared to healthy controls. Meanwhile, nine proteins i.e., ACO2, ATP5A1, ATP5B, DLD, GLUD1, GOT2, IDH2, MDH2, and UQCRC2, are found associated with metabolic pathways, while seven proteins i.e., ACO2, DLD, GLUD1, GOT2, IDH2, MDH2, and ME2 in carbon metabolism, four proteins i.e., ACO2, DLD IDH2, and MDH2 in TCA cycle, and five proteins i.e., ACO2, DLD, IDH2, MDH2, and ME2 in pyruvate metabolism (Figure 5A). These results suggest that the mitochondrial and metabolic pathways may be involved in pathologic mechanism of PBMCs from autistic children. Some other notable proteins are divided into several groups, including response to ER stress (CALR, P4HB, FLOT1, HSPA5, HSP90B1, HYOU1), protein folding in ER (CALR, HSPA5, HSP90B1), unfolded protein binding (CALR, HSP90B1, HSPA5, HSPA9, HSPD1), ER-nucleus signaling pathway and ATF6 (ATF6-alpha) activates chaperones (ALB, APOA1, CALR, HSP90B1, HSPA5), XBP1s activates chaperone genes (CALR, HSP90B1, HSPA5, HYOU1), CREB3 factors activating genes (ALB, APOA1, CALR, HSP90B1, HSPA5); endocytosis (ALB, ANXA11, APOA1, C3, CALR, FLOT1, LBP, HSP90B1, and HYOU1; Supplementary Tables S4–S6). These above mentioned proteins might be associated with ER stress and UPR, APR and inflammatory responses, suggesting that these processes might be involved in the pathophysiology of autism (Figure 5B). In addition, there are 11, 13, and 12 proteins which are associated with myelin sheath, focal adhesion, and cell adhesion (Supplementary Table S4), respectively.

### Validation of Differentially Expressed Proteins by Western Blot Analysis

**Figure 6:**
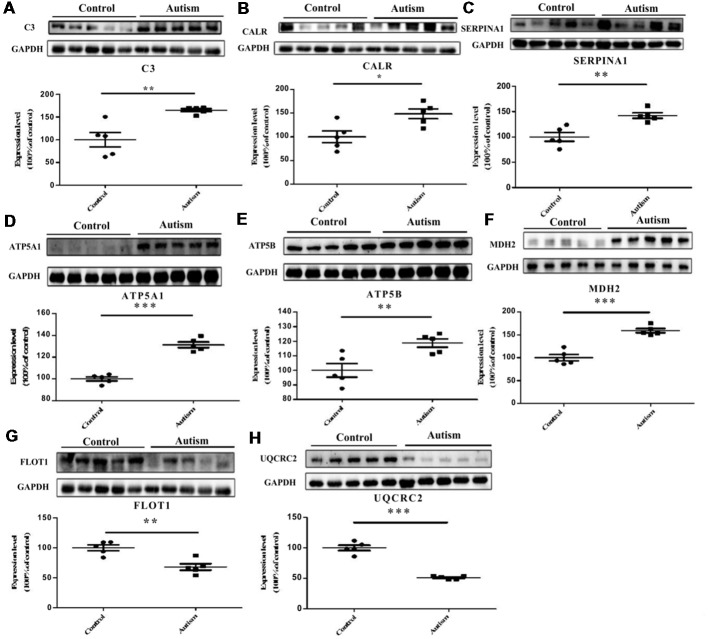
Validation of differentially expressed proteins in PBMCs of children with autism by Western blot analysis. **(A)** Complement C3 (C3). **(B)** Calreticulin (CALR). **(C)** Alpha-1-antitrypsin (SERPINA1). **(D)** ATP synthase subunit alpha, mitochondrial (ATP5A1). **(E)** ATP synthase subunit beta, mitochondrial (ATP5B). **(F)** Malate dehydrogenase, mitochondrial (MDH2). **(G)** Flotillin-1 (FLOT1). **(H)** Cytochrome b-c1 complex subunit 2, mitochondrial (UQCRC2). The level of protein expression is normalized with the mean of the controls (*n* = 5), with each bar representing the standard deviation (SD; *P* < 0.05). The upper images of Western blot analysis correspond to the lower histograms of semiquantification. *P < 0.05, **P < 0.01, ***P < 0.001.

We have selected eight proteins i.e., C3, CALR, SERPINA1, FLOT1, ATP5A1, ATP5B, MDH2, and UQCRC2 to be validated by Western blot analysis. Of which, three proteins, i.e., C3, CALR, and SERPINA1, have already been identified as differentially expressed proteins in the plasma of autistic children in our previous studies (Shen et al., 2018). These are also associated with important functions and pathways, such as metabolic pathways, ER stress, and endocytosis, etc. Consistent with the iTRAQ results, significant decrease in the expression levels of FLOT1 and UQCRC2 in the autistic patients were detected, whereas the levels of C3, CALR, SERPINA1, ATP5A1, ATP5B, and MDH2 were significantly higher than those in healthy controls (Figure 6, *P* < 0.05).

### ELISA Analysis

**Figure 7:**
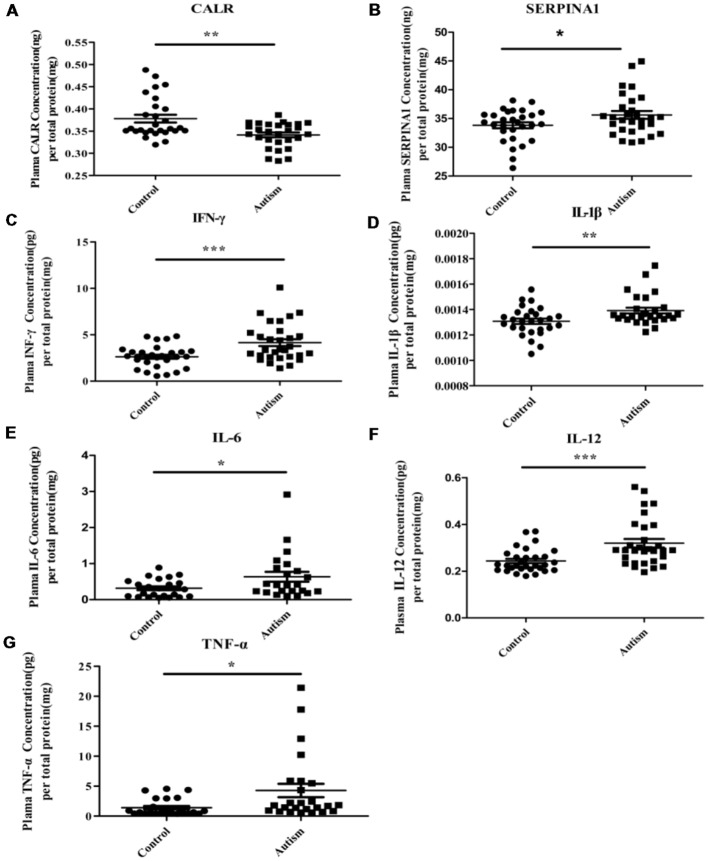
Some of differentially expressed proteins were detected in PBMCs and pro-inflammatory cytokines were detected in plasma of children with autism by enzyme-linked immunosorbent assay (ELISA) analysis. **(A)** Calreticulin (CALR). Autism and control group, *n* = 28. **(B)** Alpha-1-antitrypsin (SERPINA1). Autism and control group, *n* = 29. **(C)** Interferon-γ (IFN-γ). Autism and control group, *n* = 30. **(D)** Interleukin-1β (IL-1β). Autism and control group, *n* = 28. **(E)** Interleukin-6 (IL-6). Autism and control group, *n* = 23. **(F)** IL-12. Autism and control group, *n* = 30. **(G)** Tumor necrosis factor-α (TNF-α). Autism and control group, *n* = 25. The graphical results depict the mean ± SD, *P < 0.05, **P < 0.01, ***P < 0.001. CALR and SERPINA1 were detected in PBMCs and others pro-inflammatory cytokines were detected in plasma.

As mentioned above, three proteins (C3, CALR, and SERPINA1) have been identified in our previous study (Shen et al., 2018). Being already validated in our previous study, the expression level of C3 has not been validated here. Instead, we validated the levels of the other two proteins (CALR and SERPINA1) in the plasma by ELISA. As shown in Figures 7A,B, the results of ELISA are consistent with iTRAQ analysis. In addition, considering the potential involvement in inflammation, altered levels of pro-inflammatory cytokines have been observed in ASD individuals (Ashwood et al., 2011; Ibrahim et al., 2015; Masi et al., 2015; Xu et al., 2015). In this study, levels of IFN-γ, IL-1β, IL-6, IL-12 and TNF-α were detected in plasma (Figures 7C–G). Consistent with the previous studies (Ashwood et al., 2011; Ibrahim et al., 2015; Masi et al., 2015; Xu et al., 2015), these were significantly higher in autistic patients than in healthy controls.

## Discussion

In the present study, we have made an attempt to identify proteomic changes in PBMCs from the autistic children. Total of 41 differentially expressed proteins are found in autistic subjects as compared to healthy controls. These proteins are mainly related to mitochondrial and metabolic pathways, ER stress and protein folding, plasma lipoprotein particle levels, endocytosis, immune and inflammatory response, complement and coagulation cascades, focal adhesion, extracellular matrix, and cell-cell adherens, oxidation-reduction process, et cetera suggesting that a variety of mechanisms are associated with PBMCs in autistic children, thereby might be contributing to the development of autism.

Lot of evidence suggests that mitochondrial dysfunction may be associated with ASD. The brain of individuals with ASD may have an abnormality in carbohydrate metabolism and bioenergetics, which in turn influence some clinical symptoms, e.g., the social and cognitive deficits in ASD (Frye and Rossignol, 2011). A recent meta-analysis found that abnormal biochemical markers of mitochondrial function are relatively common in the general population of children with ASD. Moreover, it was found that a relatively high percentage of children with ASD (~5%) have mitochondrial disease (MD; Rossignol and Frye, 2012). Some studies have demonstrated decreased efficiency of electron transport chain (ETC) complex, TCA cycle and pyruvate dehydrogenase enzyme activities, as well as differences in mitochondrial gene expression in the brain tissues of individuals with autism (Rossignol and Frye, 2014). However, in this study, among the 17 differentially expressed mitochondrial proteins, 14 proteins were up-regulated in children with autism, including nine proteins associated with metabolic pathways, seven, four and five proteins involved in carbon metabolism, TCA cycle, and pyruvate metabolism, respectively (Figure 5A). Thus, our results showed that the mitochondrial protein expression patterns of PBMCs in autistic children seem to be different than previous studies on mitochondria (Frye and Rossignol, 2011; Rossignol and Frye, 2014). This implicates that the energy metabolism might be increased in the PBMCs of autistic children. Based on these findings, we may hypothesize that the PBMCs may be activated in children with autism. It may be similar to the microglial cells, whose phenotype closely resemble to monocytes/macrophages, has been reported to be activated in the brain of patients with ASD in multiple studies (Morgan et al., 2010; Rodriguez and Kern, 2011; Suzuki et al., 2013). Indeed, monocytes can serve as precursors for a number of tissue specific myeloid lineage cells including macrophages, dendritic cells, and microglia. PBMCs recruited to the central nervous system (CNS) develop into exogenously derived microglial cells or bone marrow derived microglial (BMDM) cells (Jyonouchi et al., 2011). The peripherally-activated monocytes can also enter into the brain parenchyma and release cytokines. Increased numbers of circulating monocytes have been observed in the blood and in postmortem brain tissues from ASD individuals (Sweeten et al., 2003; Vargas et al., 2005). Dendritic cells, which are important in modulating immune responses, were also found to be increased in the amygdala of individuals with ASD (Breece et al., 2013). In addition, monocytes in blood and microglia in the brain have very similar transcriptomes (Vargas et al., 2005; Morgan et al., 2010). A recent transcriptomic analysis of the autistic brain has shown the presence of two modules in the ASD brain: a neuronal module enriched for known autism susceptibility genes, and a module enriched for immune genes and glial markers (Voineagu et al., 2011). The later immune-glial module has less pronounced genetic component and thus is most likely to be either a secondary phenomenon or the result of environmental factors (Voineagu et al., 2011). Another study reported more thickened and irregular arrangement of mitochondrial cristae in PBMCs from autistic patients, implying that an activated status of mitochondria cope with an increased energy requirement in PBMCs (Siniscalco et al., 2012). Taken together, we propose that mitochondrial function may be enhanced in PBMCs and microglial cells of children with autism, whereas it might be a dysfunction in the neurons of the patients.

The mitochondrial “activation” in PBMCs of autistic children may be associated with the inflammatory and immune response. A number of studies have reported evidence of immune dysregulation and/or inflammation in individuals with ASD (Masi et al., 2015; Xu et al., 2015). Abnormal levels of various inflammatory cytokines and chemokines have been found in ASD patients in PBMCs, serum or plasma, brain tissues, and cerebrospinal fluid (CSF; Masi et al., 2015; Xu et al., 2015). For example, a series of studies found that pro-inflammatory cytokines include IFN-γ, IL-1β, IL-6, IL-12, and TNF-α were significantly higher in ASD patients, whereas anti-inflammatory cytokine IL-10 and transforming growth factor beta1 (TGF-β1) was reduced in controls (Ashwood et al., 2011; Ibrahim et al., 2015; Masi et al., 2015; Xu et al., 2015). In the brain, cytokine imbalances in ASDs may contribute directly to ASD neural dysfunction. They may influence the behavior through affecting the neurotransmitter function, neuroendocrine activity, neurogenesis, and alterations to brain circuitry (Masi et al., 2017b). On the other hand, peripheral cytokine signals are believed to access the brain (Goines and Ashwood, 2013), while changes in peripheral cytokine expression have been correlated with ASD, severity of behavioral impairments and associated symptoms (Masi et al., 2017b). In this study, several proteins associated with an acute phase response, immune and inflammatory response and IL-12 family signaling were found to enhance expression in PBMCs. Similar to previous study (Glatt et al., 2012), in transcription analyses of PBMCs, it was revealed that differentially expressed genes in ASD subjects are related to immune response, inflammation, IFN signaling and chemokine pathways. Here, five cytokines (IFN-γ, IL-1β, IL-6, IL-12, and TNF-α) were detected to be increased in plasma of autistic children. Together, these data further support that PBMCs may be activated in children with autism, which play an important role in the pathobiology of autism. Hence, supporting the view that immune system disturbances may be activated and continuously contribute to the onset of ASD in a pro-inflammatory state (Ashwood et al., 2006; Enstrom et al., 2010; Masi et al., 2017b).

It is noteworthy that several differentially expressed proteins found here relate to protein folding and unfolded protein binding. ATF6 and XBP1 activate chaperone genes, CREB3 factors activate genes and ER-nucleus signaling pathways were identified as related to these differentially expressed proteins between the case and control groups in the present study. It suggests that ER stress, UPR and APR may be involved in PBMCs from children of autism and link to the occurrence of autism (Figure 5B). The ER is a multifunctional organelle that coordinates protein folding, lipid biosynthesis, and calcium storage and release. Perturbations that disrupt ER homeostasis lead to ER stress and activation of signaling cascades termed as UPR (Chaudhari et al., 2014). The main UPR branches include PERK [PKR (dsRNA-dependent protein kinase)-like ER kinase], inositol-requiring enzyme 1 (IRE-1), and ATF-6. Under basal conditions, these specialized ER membrane-associated sensor proteins are bound by the ER chaperone BiP (also known as Grp78 or HSPA5) and are maintained in an inactive state (Zhang and Kaufman, 2008; Chaudhari et al., 2014). Accumulation of unfolded/misfolded/mutated proteins in the ER lumen activates adaptive UPR mechanisms through release of BiP from the sensor proteins and initiation of specific cellular responses. All these events points towards the restoration of ER homeostasis (Chaudhari et al., 2014). The released ATF6 acts as a transcription factor, which travels to the nucleus and binds to ER-stress response elements (ERSE) and regulates gene expression of proteins involved in ER stress and UPR, along with Grp78, PDIs, and activate transcription of major APR genes. In response to ER stress, IRE1α autophosphorylates, thereby activates its RNase activity. It then splices XBP1 mRNA to splice XBP1 mRNA, which code for a transcription factor XBP1s that translocates to the nucleus and regulates genes involved in UPR and ER-associated degradation (ERAD; Zhang and Kaufman, 2008; Chaudhari et al., 2014). Likewise, CREB3 proteins were also identified to mediate the APR (Fox and Andrew, 2015). Thus, our results suggest that activation of ATF6, XBP1s and CREB3 may be involved in ER stress, UPR, and APR in autistic PBMCs (Figure 5B). Interestingly, a recent study showed a significant increase in the mRNA levels of ATF4, ATF6, PERK, XBP1, CHOP, and IRE1 in the middle frontal gyrus of ASD subjects (Crider et al., 2017). Another study showed that the activation of UPR specifically regulated glutamate neurotransmission in the cerebellum of a mouse model of autism (Trobiani et al., 2018). Taken together, these results support that ER stress and UPR may be involved in pathogenesis of ASD.

In addition, ATF6 activation can induce inflammation *via* promoting the transcription of genes for cytokines, chemokines, and other pro-inflammatory molecules by nuclear factor-κB (NF-κB) pathway (Zhang and Kaufman, 2008; Chaudhari et al., 2014). IRE1α can activate the JNK-AP1 and NF-κB pathways and induces the expression of inflammatory genes (Zhang and Kaufman, 2008; Chaudhari et al., 2014). Thus, these may have a correlation between ER stress and inflammatory responses in the PBMCs of autistic children and pathogenesis of autism. Indeed, compared with healthy controls, NF-κB DNA binding activity was found elevated in peripheral blood samples of children with autism (Naik et al., 2011), and increased NF-κB expression levels were also found in post-mortem samples of orbitofrontal cortex from autistic patients, suggesting the activation of NF-κB pathways (Young et al., 2011). Likewise, five proteins i.e., FLOT1, FLOT2, HSP90B1, HSPD1, LBP are related to the TLR signaling pathway in this study, suggesting that it may be associated with inflammatory processes of PBMCs from autistic children. Recent studies have shown that TLR control of immune response is altered in monocyte cultures from children with ASD (Enstrom et al., 2010) and associated microglial cells (Réus et al., 2015). Activation of TLR signaling can trigger NF-κB-mediated inflammatory response. The activation of inflammatory pathways result in the release of pro-inflammatory cytokines such as IL-1β, IL-6, IL-12, and TNF-α, IFN-γ, et cetera, which then amplify the inflammatory reactions (Lawrence, 2009; Enstrom et al., 2010). Hence, it can be proposed that ER stress, UPR and ATF6 pathway, IRE1α pathway, TLR signaling pathway and pro-inflammatory cytokines might be involved in inflammatory processes of PBMCs from autistic children. Besides ASD, ER stress and UPR have been observed in a variety of diseases including metabolic disease, neurodegenerative disease, inflammatory disease, cancer, and autoimmunity (Wang and Kaufman, 2012). The induction of ER stress and activation of the UPR *in vivo* is attributable to both intrinsic and extrinsic factors (Wang and Kaufman, 2016). For autism, autism-associated mutations in several genes have been shown to affect protein folding and lead to activation of the UPR and ER stress (Fujita et al., 2010; Ulbrich et al., 2016). It may also be due to oxidative stress, autoimmune system, and inflammatory factors that need to be further clarified.

Additionally, inflammation is a controlled response of the host to infection or injury that involves various molecular, cellular and physiological changes and is coordinated primarily by specific cell adhesion molecules and chemo-attractants (Ghosh et al., 2014). Circulating monocytes then migrate across the endothelium to gain access to the site of infection or injury (Ghosh et al., 2014). Here, some proteins involved in cell adhesion, focal adhesion, cell-substrate junction, adherens junction, and anchoring junction, may be related to the recruitment and migration of PBMCs from autistic patients, thereby contributing to inflammatory reaction in the cells. In addition, the inflammatory processes are accompanied by phagocytic reactions. Endocytosis plays an important role in inflammation. In this study, nine proteins associated with endocytosis were found to be altered between autistic subjects, implying that they may be enhanced in PBMCs of autistic children.

Furthermore, these proteins may serve as candidate biomarkers for the diagnosis of autism. However, the mechanisms associated with these proteins such as inflammation, protein misfolding and alteration of mitochondrial activity are presumably common responses to many internal and external challenges, thereby affecting their specificity as a diagnostic marker. Nevertheless, very recently, two review studies on autism proteomics and biomarkers highlighted the close relationship between these mechanisms and ASD (Abraham et al., 2019; Shen et al., 2019). They may be interacted with each other, and related to the occurrence and development of these diseases, and may be primary or secondary phenomena (Abraham et al., 2019). Given the complexity of ASD pathogenesis, a panel of proteins rather than a single protein could be a more powerful approach to diagnose this disease. Besides, discovering and detecting parallel change between CNS and peripheral samples is one of the strategies for finding diagnostic markers for ASD (Hayashi-Takagi et al., 2014; Shen et al., 2019). Indeed, various abnormalities in immunological markers or immune functions, including CSF or brain tissue-associated immune abnormalities have been a recurring theme in ASD research (Abraham et al., 2019). In addition, as mentioned above, ER stress has been observed in the brains of autistic children (Crider et al., 2017), and UPR activation has also been observed in the cerebellum of a mouse model of autism (Trobiani et al., 2018). Therefore, these proteins still have the potential to become diagnostic markers. Moreover, here, three proteins, i.e., C3, CALR, and SERPINA1, were simultaneously changed in blood and cells of autistic subjects, suggesting that analyses of their expression levels both in the plasma and PBMCs in case and control groups could increase the specificity of the marker. Interestingly, the expression of CALR is reversed in the current study of PBMCs and plasma from our previous study (Shen et al., 2018). It might reflect differences in the utility of each biological specimen for diagnosis. Complement protein C3 has also been detected to be increased in plasma of children with ASD in a previous study (Corbett et al., 2007). The complement system, a complex protein network initially identified as part of the innate immune system, is an essential regulator of cell and tissue homeostasis. C3 is the central component of the complement system which induces inflammatory, immunomodulatory and metabolic responses. In addition, C5 was detected to be increased in autistic children in our previous study (Shen et al., 2018). Besides, three proteins i.e., A2M, SERPINA1, and SERPIND1 were also associated with complement and coagulation cascades in the present study, indicating that complement system and coagulation cascades may be involved in the pathogenesis of autism.

In conclusion, to the best of our knowledge, this is the first study to investigate PBMCs from children with autism using iTRAQ technique, and 41 differentially expressed proteins were identified between the case and control groups. Of which 17 mitochondrial proteins associated with different metabolic pathways were increased in autistic children. Some of them were verified and five pro-inflammatory cytokines were found to be increased in the plasma of autistic children. The results showed that ER stress and the UPR, APR and inflammatory response were associated with PBMCs from autistic children, and they may be induced by activation of ATF6, XBP1s, and CREB3. ATF6, IRE1α and TLR signals trigger the activation of NF-κB that may be responsible for the inflammatory response in the PBMCs of autistic children. The recruitment, migration, and endocytosis of PBMCs may also be involved in the inflammatory process. Our results suggest that the PBMCs from autistic children might be activated and are linked to the occurrence of autism. These proteins may serve as candidate proteins for the diagnosis of autism and needs to be studied further. Meanwhile, it is necessary to point out that the present study is a preliminary investigation. These differentially expressed proteins need to be further validated by using Western blot analysis and/or ELISA assay, and a large scale validation and a non-mixed sample strategy for proteomics analysis are also required.

## Ethics Statement

The research was approved by Human Research Ethics Committees of Maternal and Child Health Hospital of Baoan and complies with the guidelines of the Helsinki Declaration. Written informed consent for study participation was obtained from parents of children.

## Author Contributions

LS and CF conceived and designed the experiments, analyzed the data and drafted the manuscript. KZ, YC, JK, XC, JL, CL, JI, and YZ performed the experiments and helped to revise the manuscript. YG carried out the clinical diagnosis. CF, YG, and WW recruited patients, and collected the blood samples and helped to revise the manuscript. All authors read and approved the final manuscript.

## Conflict of Interest Statement

The authors declare that the research was conducted in the absence of any commercial or financial relationships that could be construed as a potential conflict of interest.

## Footnotes

^1^http://www.omicsbean.cn/

^2^http://david.abcc.ncifcrf.gov/

## Supplementary Material

FIGURE S1 Figures 4A,B are presented separately.

TABLE S1 Gradient of mobile phase and run time of nanoLC-MS/MS analysis.

TABLE S2 List all quantitative proteins in the peripheral blood mononuclear cells (PBMCs) in this study.

TABLE S3 Differentially expressed proteins identified from the peripheral blood mononuclear cells (PBMCs) of autistic children and healthy controls.

TABLE S4 Differentially expressed proteins associated with gene ontology (GO) and signal transduction pathways in DAVID analysis.

TABLE S5 Differentially expressed proteins associated with gene ontology (GO) by using ClueGO analysis.

TABLE S6 Differentially expressed proteins associated with signal transduction pathways by using ClueGO analysis.

## Abbreviations

APR: acute-phase response
ASD: Autism spectrum disorder
ELISA: enzyme-linked immunosorbent assay
ER stress: endoplasmic reticulum
MS: mass spectrometry
iTRAQ: isobaric tags for relative and absolute quantitation
PBMCs: peripheral blood mononuclear cells
TLR: toll-like receptor
UPR: unfolded protein response.
