# Supplementary figures and images for: Proteomics Study of Peripheral Blood Mononuclear Cells (PBMCs) in Autistic Children
Source: Front Cell Neurosci. 2019 Mar 19;13:105. doi: 10.3389/fncel.2019.00105 (PMC6433831; doi:10.3389/fncel.2019.00105)

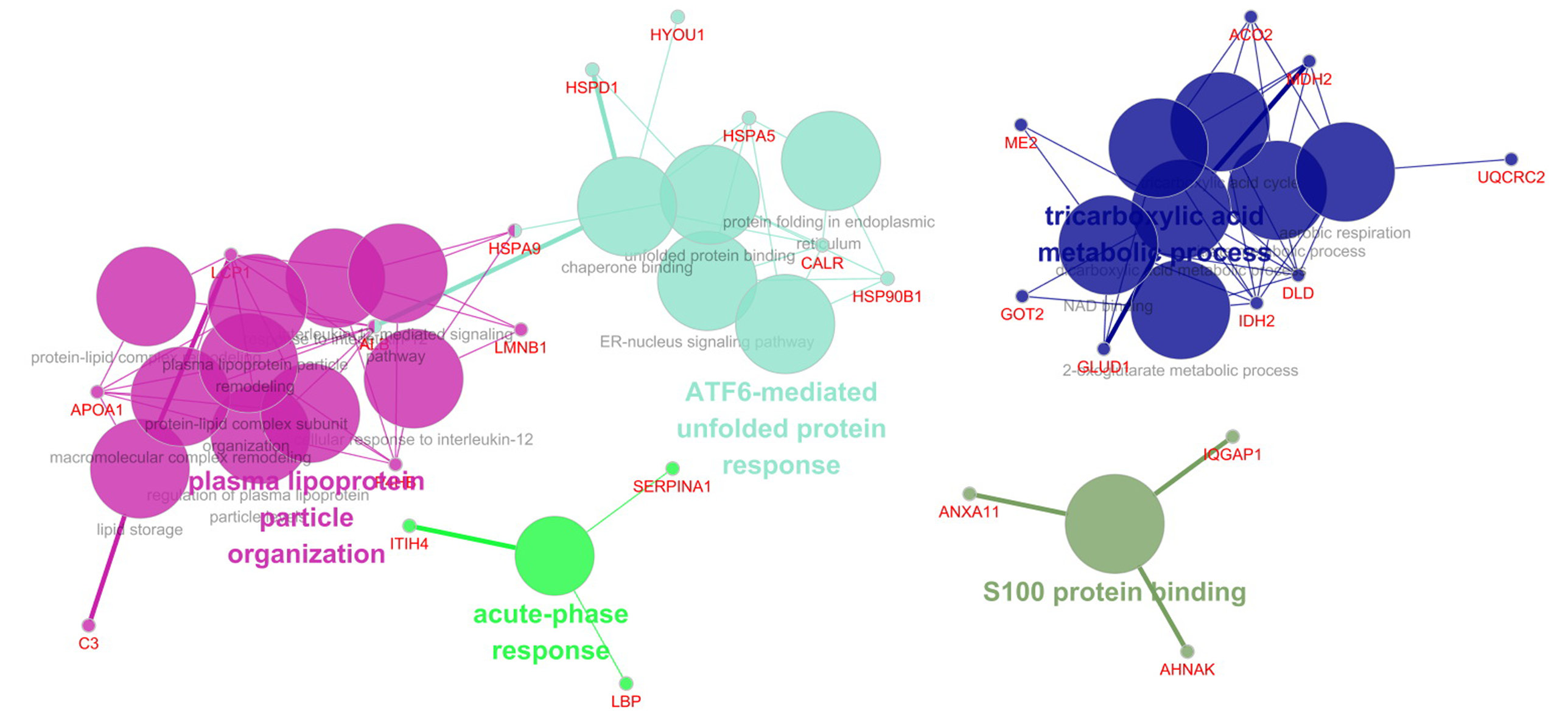

Supplement: FIGURE S1 — Figures 4A,B are presented separately. [file Image_1.TIF]
