# Supplementary material for: Proteomics Study of Peripheral Blood Mononuclear Cells (PBMCs) in Autistic Children
Source: Front Cell Neurosci. 2019 Mar 19;13:105. doi: 10.3389/fncel.2019.00105 (PMC6433831; doi:10.3389/fncel.2019.00105)
Supplement: TABLE S1 — Gradient of mobile phase and run time of nanoLC-MS/MS analysis. [file Table_1.DOCX]

**Table S1**. Gradient of mobile phase and run time of nanoLC-MS/MS analysis.

| Time (min) | B%（1-4*） | B%（5-10*） |
| --- | --- | --- |
| 0 | 5 | 5 |
| 60 | 25 | 26 |
| 80 | 48 | 40 |
| 80.02 | 80 | 80 |
| 86 | 80 | 80 |
| 86.02 | 5 | 5 |
| 90 | 5 | 5 |

*After labeling, peptides were eluted and combined into 10 groups.
